# Differential Expression Profiles of Orphan Nuclear Receptors (NR4A) and N-myc Downstream-Regulated Gene Family (NDRG) in Patients with Inflammatory Bowel Disease

**DOI:** 10.3390/ijms27114769

**Published:** 2026-05-26

**Authors:** Gabriela Fonseca-Camarillo, Janette Furuzawa-Carballeda, Diana Aguilar-León, Rafael Barreto-Zuñiga, Braulio Martínez-Benítez, Jesús K. Yamamoto-Furusho

**Affiliations:** 1 Inflammatory Bowel Disease Clinic, Department of Gastroenterology, Instituto Nacional de Ciencias Médicas y Nutrición Salvador Zubirán, Mexico City 14080, Mexico; gabrielafaster@gmail.com; 2 Department of Immunology, Instituto Nacional de Cardiología, Ignacio Chávez, Mexico City 14080, Mexico; 3 Department of Experimental Surgery, Instituto Nacional de Ciencias Médicas y Nutrición Salvador Zubirán, Mexico City 14080, Mexico; jfuruzawa@gmail.com; 4 School of Medicine, Universidad Panamericana, Mexico City 03920, Mexico; 5 Department of Pathology, Instituto Nacional de Ciencias Médicas y Nutrición Salvador Zubirán, Mexico City 14080, Mexico; aguilarleon@hotmail.com (D.A.-L.); brauliomb77@yahoo.com.mx (B.M.-B.); 6 Department of Endoscopy, Instituto Nacional de Ciencias Médicas y Nutrición Salvador Zubirán, Mexico City 14080, Mexico; barretozu@yahoo.com

**Keywords:** NDRGs, NR4A2, IBD, receptors, inflammation

## Abstract

NDRG1 protein engages with the orphan nuclear receptor NR4A1, effectively suppressing the transcriptional activity of NF-κB and influencing the inflammatory response. However, the specific roles of the NDRG family and NR4A transcription factors in inflammatory bowel disease (IBD) remain poorly defined, particularly regarding potential differential mechanisms between ulcerative colitis (UC) and Crohn’s disease (CD). We hypothesize that NDRG–NR4A interactions are differentially regulated in UC versus CD, contributing to disease-specific modulation of NF-κB signaling and inflammatory responses. Therefore, the aim was to analyze gene and protein expression of both protein families (NDRGs: NDRG1, NDRG2, NDRG3, and NDRG4; and NR4A: NR4A1, NR4A2, and NR4A3), their contributions to UC and CD, and their association with disease severity. In this cross-sectional and comparative study, we assess gene and protein expression of NR4A and NDRG1-4 in 38 UC patients, 10 CD patients, and 18 controls. Gene and protein expression levels were measured by RT-PCR (mucosa) and immunohistochemistry (colonic tissue), respectively. The colonic mucosa from remission UC patients showed upregulation of *NDRG2* and the nuclear receptor genes *NR4A1-3* compared with controls. *NDRG4* was upregulated in active UC patients compared with controls. *NDRG1* was downmodulated in active and remission UC patients compared with controls. All differences were statistically significant (*p* < 0.05). Decreased *NR4A2* gene expression was associated with high-sensitivity C-reactive protein (*p* = 0.030) and erythrocyte sedimentation rate levels (*p* = 0.001). Our results provide the first evidence of differential alterations in the NDRG–NR4A axis in UC and CD, which could modulate NF κB signaling and the inflammatory profile differently in each disease, opening the possibility of new therapeutic options.

## 1. Introduction

Inflammatory bowel disease (IBD) encompasses both ulcerative colitis (UC) and Crohn’s disease (CD), which are classified as chronic inflammatory disorders linked to abnormal immune responses [1]. UC primarily affects the colonic mucosa, whereas CD can involve any segment of the gastrointestinal tract [1,2]. In recent years, Mexico has witnessed a significant rise in IBD prevalence, increasing from 0.30 to 1.83 cases per 100,000 person-years from 2000 to 2015 (*p* = 0.05). This surge also reflects an incidence increase of 5.9-fold for IBD overall, with specific increases of 5.3-fold for UC and 9.5-fold for CD. The data indicate that peak prevalence occurs in individuals aged between 20 and 40 years; additionally, hospitalized patients showed two distinct peaks: one over the age of 60 and another within the age range of 20–40 years [3,4]. Understanding the disease’s pathophysiology is crucial for identifying potential therapeutic targets aimed at curbing the rising prevalence and incidence rates of IBD.

The N-myc downstream-regulated gene family comprises *NDRG1*, *NDRG2*, *NDRG3*, and *NDRG4*, all of which belong to the alpha/beta hydrolase superfamily. The cytoplasmic protein encoded by these genes is implicated in stress responses, hormonal signaling, cell growth, and differentiation [5,6]. Notably, NDRG1 plays a significant role in allergic reactions and anaphylaxis, as well as in defending against bacterial infections, while also promoting inflammation and wound healing processes. In mast cells specifically, NDRG1 expression increases during maturation and facilitates rapid degranulation in response to various stimuli [7].

Research has shown that NDRG1 is upregulated during clonal T-cell anergy via EGR2 signaling, eliminating the need for stimulation to inhibit further T-cell reactivation through TCR/CD28 signaling pathways [7]. While less is known about other members of the NDRG family concerning immune responses, some evidence suggests that they may negatively regulate WNT signaling pathways by suppressing CTNNB1-mediated transcriptional activation of target genes like CCND1, thus potentially functioning as tumor suppressors through their involvement in apoptosis, autophagy, and PI3K/Akt signaling pathways [8].

An association has been identified between nuclear factor (NF)-κB translocation and NR4A transcription factor activation, particularly in biological processes such as apoptosis regulation, cell proliferation control, angiogenesis promotion, DNA repair mechanisms, and fatty acid metabolism regulation [9]. The NR4A family includes three members: NR4A1 (NGFI-B/Nur77/TR3), NR4A2 (NURR1/HZF-3/RNR1), and NR4A3 (NOR1/MINOR/TEC). These receptors are involved in diverse functions, such as regulating energy metabolism, modulating apoptotic processes, controlling activities related to the hypothalamic–pituitary axis and reproductive functions, and mediating pro-inflammatory responses [10].

Gene expression analysis has revealed that NR4A receptor levels are present in human tissues exhibiting chronic inflammation, as well as in murine models induced with various pathologies, including atherosclerosis, arthritis, and psoriasis [11,12,13,14]. Although no endogenous ligands have been identified for NR4A receptors, pharmacological agents such as 6-mercaptopurine or derivatives of bis(indolyl)methane can effectively modulate their activity [11,15,16,17].

NR4A receptors exhibit constitutive expression patterns controlled by factors including transcriptional regulation, post-translational modifications, protein interactions, and subcellular localization dynamics [18,19,20,21,22]. NF-κB acts as a vital regulator influencing NR4A gene expression within myeloid lineage cells. Research highlights the feedback regulatory roles played by NR4As in controlling NF-κB activity, along with managing pro-inflammatory gene expression primarily through repression mechanisms that target NF-κB transcriptional activity [13].

Numerous studies have affirmed that the involvement of the NR4A receptor significantly influences monocyte/macrophage differentiation. Loss-of-function results lead to exacerbated hyperinflammatory cellular responses, attributed mainly to uncontrolled NF-κB signaling pathways, alongside reductions in functional p65 activity observed under these conditions [23,24,25,26].

Importantly, however, it should be noted that NR4A1 can promote NF-kB activity following stimulation via murine macrophage IKKi action mechanisms [26]. Discoveries from earlier investigations into how specific family members are involved in apoptosis modulation are ongoing, especially in inflammatory contexts. Despite the lack of studies examining interactions between NR4As and NDRGs within IBD patient populations, this regulatory protein set may represent new strategies for treating IBD. Understanding their engagement has become paramount for elucidating the origins and related development aspects. We hypothesize that altered expression of NDRG and NR4A, and differentially regulated interactions in UC versus CD, contribute to the modulation of NF-κB signaling and consequently to inflammatory responses.

In the present study, we aim to analyze gene and protein expression in both families (NDRG1-4 and NR4A1-3) and their contributions across UC and CD patient groups. Finally, these expressions are associated with disease severity levels across the examined cohorts.

## 2. Results

In the study, we included a total of 48 consecutive patients diagnosed with IBD, confirmed through histopathological methods. Specifically, there were 38 UC cases—20 active and 18 in remission—alongside 10 active CD subjects and 18 controls devoid of intestinal inflammation. In our cohort, patients with CD presented predominantly with moderate-to-severe clinical activity, allowing comparison with UC patients who showed a similar inflammatory profile. Patients with active UC had a mean age of 40.3 years, while patients in remission had a mean age of 40.2 years. Patients with active CD had a mean age of 41.8 years.

Most patients with active UC had an intermittent clinical course characterized by continuous disease activity, whereas patients with UC in remission initially presented with active disease followed by an inactive phase. Patients with CD exhibited both intermittent and continuous patterns of disease activity.

Patients with active UC primarily showed (E1) disease extension, corresponding to proctitis, whereas patients in remission presented left-sided colitis (E2) and pancolitis (E3). Most patients with active UC exhibited extraintestinal manifestations, whereas those with UC in remission did not. Detailed characteristics are mentioned in Table 1.

### 2.1. Relative Gene Expression of NDRGs (NDRG1, NDRG2, NDRG3, and NDRG4) in UC Patients and Controls

**Figure 1:**
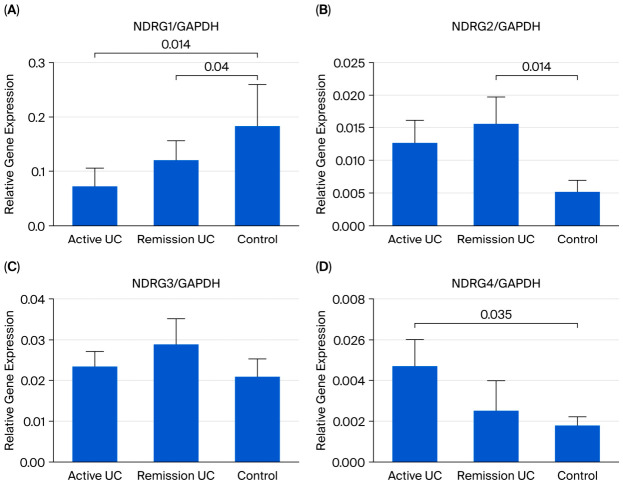
Gene expression of the *NDRG* family (*NDRG1*, *NDRG2*, *NDRG3*, and *NDRG4*) in the rectal mucosa of UC patients and controls. Units are in terms of relative gene expression. Bars show the means ± standard error of the mean for transcript levels of (**A**) *NDRG1*, (**B**) *NDRG2*, (**C**) *NDRG3*, and (**D**) *NDRG4* relative to *GAPDH* as the housekeeping gene, determined by differences among groups and assessed by Dunn’s test. A *p* value of <0.05 was considered significant.

*NDRG1* gene expression levels in the active and remission colonic mucosa of UC patients were decreased compared to the control group (*p* = 0.014 and 0.04, respectively; Figure 1A), suggesting that the gene downregulation could be attributed to the disease and inflammatory conditions.

*NDRG2* in the remission UC group was upregulated at a statistically significant level (*p* = 0.014), suggesting that the increased gene levels might be related to apoptosis regulation.

*NDRG4* was upregulated in the active UC group compared to the control group (*p* = 0.035; Figure 1D), suggesting that it could impact the clinical outcome due to its suppressive function.

*NDRG3* did not differ significantly between active and remission UC or the control group (Figure 1B,C).

### 2.2. Protein Expression of NDRG Family (NDRG1, NDRG2, and NDRG4) in Intestinal Tissue from Patients with Active UC and CD

The NDRG1-, NDRG2-, and NDRG4-expressing cells were identified in magenta (cells stained with red chromogen and depicted by magenta arrows), and CD123+ plasmacytoid dendritic cells were identified as brown (cells stained with diaminobenzidine and depicted by brown arrows). This cell population has been previously identified as a regulatory dendritic cell subpopulation capable of modulating the pro-inflammatory response. The double-positive NDRG and CD123 cells were identified in burgundy (dotted burgundy arrows depict NDRG+/CD13+ cells). Intestinal tissue from patients with UC and CD exhibited abundant inflammatory infiltrates, predominantly mononuclear cells, extending from the serosal layer to the mucosa. The infiltrates were more abundant in the epithelium. Increased lamina propria cellularity, basal plasmacytosis and lymphoid aggregates, and cells morphologically compatible with neutrophils were observed. Significant mucosal destruction was observed in all cases. Alterations in the architecture between crypts, including branching, crypt distortion, atrophy, and surface irregularity, were also observed.

In patients with ulcerative colitis, immunoregulatory NDRG1+/CD123+ cells were observed only in the muscular layer, at a proportion similar to that in the control group. In contrast, these double-positive cells were absent in the mucosa, submucosa, and serosa.

**Figure 2:**
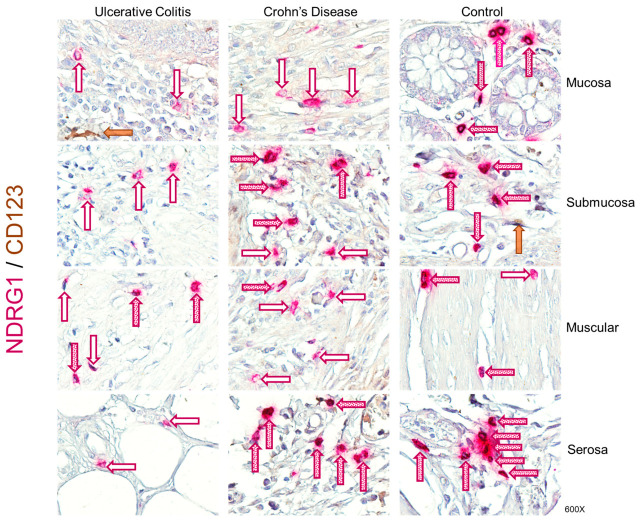
NDRG1 and CD123 localization in intestinal tissue from UC, CD, and non-inflamed controls. Magenta arrows show NDRG1+ cells, brown arrows depict CD123+ cells, and dotted burgundy arrows mark NDRG1/CD123 double-positive cells in mucosa (*file 1*), submucosa (*file 2*), muscular (*file 3*), and serosa (*file 4*) layers from ulcerative colitis (**left column**), Crohn’s disease (**middle column**), and control group (**right column**). Original magnification, 600×.

In patients with Crohn’s disease, NDRG1+/CD123+ cells were absent in the mucosa, whereas in the remaining layers, a proportion similar to that in the control group was observed (Figure 2).

**Figure 3:**
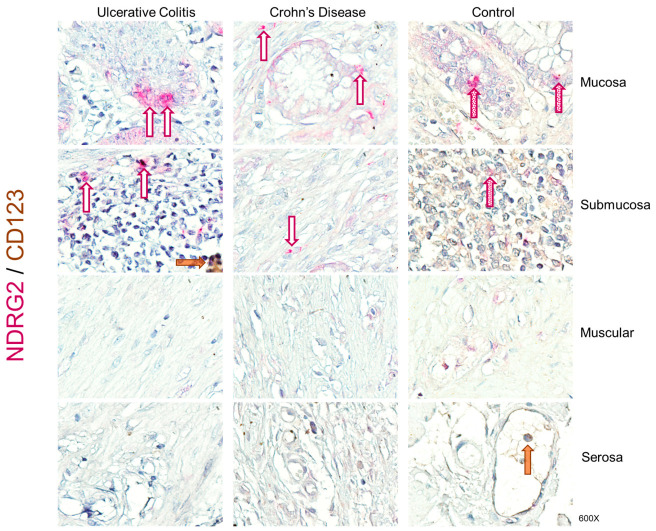
NDRG2 and CD123 detection in the intestinal tissue from UC, CD, and non-inflamed controls. Magenta arrows show NDRG2+ cells, brown arrows depict CD123+ cells, and dotted burgundy arrows mark NDRG2/CD123 double-positive cells in mucosa (*file 1*), submucosa (*file 2*), muscular (*file 3*), and serosa (*file 4*) layers from ulcerative colitis (**left column**), Crohn’s disease (**middle column**), and control group (**right column**). Original magnification, 600×.

Regarding NDRG2/CD123 double-positive cells (plasmacytoid dendritic cell populations with tolerogenic function), they were detected only in the mucosa and submucosa of the control group. The absence of this subpopulation was observed across all layers of active UC and active CD patients and was correlated with disease activity, suggesting that its absence is involved in disease development (Figure 3).

**Figure 4:**
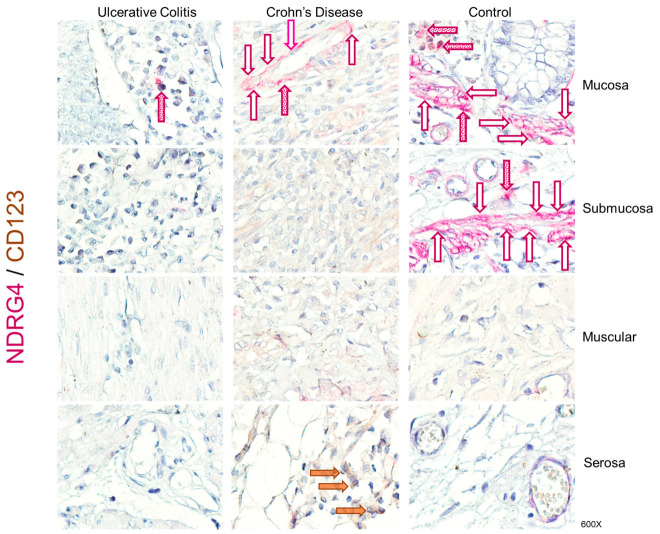
NDRG4 and CD123 in the intestinal tissue from patients with UC, CD, and non-inflammatory controls. Magenta arrows show NDRG4+ cells, brown arrows depict CD123+ cells, and dotted burgundy arrows mark NDRG4/CD123 double-positive cells in mucosa (*file 1*), submucosa (*file 2*), muscular (*file 3*), and serosa (*file 4*) layers from ulcerative colitis (**left column**), Crohn’s disease (**middle column**), and control group (**right column**). Original magnification, 600×.

Finally, NDRG4/CD123 double-positive cells were decreased in the mucosa of active UC and CD tissues compared to the control group. The absence of this subpopulation was observed in the submucosa, muscular, and serosa layers of active UC and CD patients, suggesting a lack of tolerogenic mechanisms in active disease (Figure 4).

### 2.3. Relative Gene Expression of NR4A Transcription Factors (NR4A1, NR4A2, and NR4A3) in UC Patients and Controls

**Figure 5:**
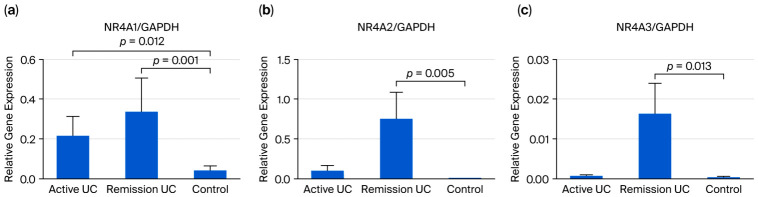
Gene expression of the *NR4A* family (*NR4A1*, *NR4A2*, and *NR4A3*) in the rectal mucosa of UC patients and controls. Bars show means ± standard error of the mean for transcript levels of (**a**) *NR4A1*, (**b**) *NR4A2*, and (**c**) *NR4A3* relative to *GAPDH* as the housekeeping gene, determined by differences among groups and assessed by Dunn’s test, with *p* values presented in the figure.

To identify the regulation of NR4A transcription factor expression, we explored the relative gene expression of the family members in the rectal mucosa of UC patients and controls. The expression of *NR4A1*, *NR4A2*, and *NR4A3* genes was increased in the colonic mucosa of UC patients in remission compared to the controls (*p* = 0.01, *p* = 0.005, and *p* = 0.013, respectively; Figure 5). Decreased *NR4A2* gene expression was associated with hs-C-reactive protein (*p* = 0.030) and erythrocyte sedimentation rate (*p* = 0.001), suggesting that reduced *NR4A2* expression may correlate with increased systemic inflammation and clinical disease activity. These findings suggest that NR4A2 may regulate immune and inflammatory pathways involved in IBD pathogenesis and may serve as a potential biomarker of disease activity.

## 3. Discussion

To our knowledge, this is the first study to characterize the NDRG protein family and NR4A transcription factors in intestinal tissue from IBD patients and to infer their potential role in inflammation regulation.

The N-myc-regulated gene (NDRG) family acts as adaptor proteins that modulate interactions between kinases and phosphatases (such as PP2A), thereby affecting the phosphorylation of receptors and intracellular signaling molecules, including NF-κB, AKT, STAT3, and TGF-β. They are actively involved in regulating inflammation, primarily by modulating cellular stress, immune signaling, and tissue. They often act as “sensors” that are activated by hypoxia or inflammatory stress [27]. NDRG1 upmodulates inflammation by interacting with the nuclear receptor Nur77 (a suppressor of vascular inflammation), inhibiting its anti-inflammatory activity and thereby activating pro-inflammatory pathways such as NF-κB. It induces the expression of adhesion molecules (VCAM-1 and ICAM-1) and cytokines (IL-6 and IL-8), facilitating leukocyte recruitment [28]. NDRG1 subcellular expression was predominantly on the nuclear membrane and cytoplasm, with lesser amounts on the cellular membrane and adherent junctions. The ubiquitous localization of this protein also suggests pleiotropic functions, including a role in CD123-positive cells.

NDRG1-immunoreactive cells were abundant and predominantly detected in epithelial cells, goblet cells, and submucosal and serosal cells in control subjects, indicating constitutive expression and maintenance of tolerance. Interestingly, NDRG1 expression in colonic mucosa from UC and CD was almost absent compared with controls, suggesting a lack of downregulation of inflammatory mechanisms in the intestinal barrier. Reinforcing this finding, a previous bioinformatic analysis identified NDRG1 as a potential candidate gene in the progression and development of IBD [29]. NDRG1/CD123-expressing cells in active CD were higher than in active UC, indicating that CD had a less severe inflammation.

NDRG2 generally participates in the protection of the intestinal barrier. Synthesis of NDRG2 enhanced the interaction of the E3 ligase FBXO11 with the Snail protein (the repressor of E-cadherin) to promote Snail degradation by ubiquitination and maintain E-cadherin expression [30]. Its deficiency reduces E-cadherin expression, increasing permeability and promoting colitis [30].

It is worth noting that, in our study population, the mucosa of patients with active UC and CD lacked NGDR2+/CD123+ cells, suggesting a loss of tolerance mechanism and protective mechanisms of the gastric mucosa and, consequently, a more severe form of the disease due to damage to the intestinal barrier.

NDRG3, also regulated by hypoxia, has been implicated in cellular homeostasis; it interacts with pathways similar to those of its homologs.

Last but not least, NDRG4 and its associated pathways, particularly through neuregulin-4 (NRG4)/ErbB4 signaling, play a fundamental role as negative regulators of inflammation, acting as a feedback mechanism to limit the production of pro-inflammatory cytokines (TNF-α, CXCL1, and IL-1β) in macrophages and tissues and protecting against chronic tissue damage. Deletion of its receptor or ligand leads to increased severity in colitis models, indicating its protective role [31]. The expression pattern of NDRG4 in plasmacytoid dendritic cells was very similar to that of NDRG2, which was decreased in the mucosa of patients with active UC and CD compared to controls. In the internal layers—submucosa, muscular, and serosa—no NDRG4+ cells were detected. Interestingly, perivascular inflammatory infiltrates from patients with active IBD were negative for NDRG4 cells, corroborating a previous study demonstrating that NDRG4 expression in colorectal cancer is frequently inactivated by promoter methylation [32].

In another study aimed at assessing the noninvasive early diagnosis of CRC by examining NDRG4 gene promoter methylation in peripheral blood mononuclear cells (PBMCs), the methylation rate for NDRG4 was 38.8% in CRC patients and 12.23% in healthy controls (*p* < 0.001) [33].

Importantly, its role in UC has been poorly characterized; our findings provide novel evidence that NDRG4 is induced in the inflamed colonic mucosa of patients with active UC, suggesting a previously unrecognized role for NDRG4 in the inflammatory response and its potential involvement in mucosal immune regulation.

However, little is known about the role of NDRG4 in the inflammatory response; these findings demonstrate that an inflammatory stimulus induces NDRG4 transcription in patients with active UC.

Further studies on NDRGs and NR4A1 proteins in the gut mucosal immune response and their mechanism in human IBD are needed to confirm and support their inflammatory role.

Our study is limited by a small sample size, and the use of bulk RNA from whole intestinal biopsies may have reduced our ability to detect cell-specific changes in orphan nuclear receptor (NR4A) and NDRG expression.

Our results shed further light on the preponderant role of NDRGs in regulating NR4A1 proteins as a tolerogenic mechanism in IBD and warrant an in-depth study to evaluate the clinical relevance of these findings. They may be used as specific therapeutic targets in IBD treatment. However, further experiments are still needed to confirm our results.

In conclusion, the gene expression of transcription factors NDRG2, NDRG3, NR4A1, NR4A2, and NR4A3 is upregulated in patients in remission. Low NR4A2 levels are associated with elevated CRP and ESR. These findings suggest an immunomodulatory role for the NDRG protein family and NR4A1 transcription factors in patients with UC.

## 4. Materials and Methods

### 4.1. Patients

This study was a cross-sectional and comparative analysis involving 38 patients with UC and 10 with Crohn’s disease. They were recruited from October 2021 to May 2023. Mucosal disease activity was evaluated using the novel Yamamoto-Furusho index for UC (Remission: 0 to 3 points, Mild Activity: 4 to 6 points, Moderate Activity: 7 to 12 points, and Severe Activity: 13 to 18 points) [34]. Key variables assessed included age, gender, and extraintestinal manifestations. The Yamamoto-Furusho index evaluated (*i*) bloody bowel movements per day: increase from 0 (usual) to 3 (≥6); (*ii*) hemoglobin (g/dL): decrease from 0 (≥12) to 3 (<10); (*iii)* C-reactive protein (hs-CRP): increase from 0 (≤0.20) to 3 (>1.0); (*iv*) albumin (g/dL): decrease from 0 (≥3.5) to 3 (<3.0); (*v*) endoscopic findings (Mayo): from 0 (normal) to 3 (spontaneous bleeding/ulceration); and (*vi*) histological findings: from 0 (normal) to 3 (severe infiltration/crypt destruction). Clinical progression was categorized into (*i*) sustained remission for over five years (initially active, followed by long-term remission), (*ii*) intermittent activity (more than 2 relapses annually), and (*iii*) chronic continual activity (persistent symptoms despite standard medical treatment). Patients with a diagnosis of autoimmune diseases and/or cancer were excluded. The control group consisted of 18 individuals who underwent colonoscopy due to weight loss or anemia but exhibited no signs of intestinal inflammation, either clinically or endoscopically, nor did they have any systemic diseases such as cancer or autoimmune disorders. All enrolled participants with IBD were from the Inflammatory Bowel Disease Clinic at the Instituto Nacional de Ciencias Médicas y Nutrición Salvador Zubirán. They provided their free and informed consent for participation.

### 4.2. Gene Expression Analysis

Quantification of gene expression for NDRG family members was conducted following established methodologies [35,36,37]. A total of 66 colonic mucosal biopsies were collected and stored in cryovials containing RNA preservative (RNA later^®^, Thermo Fisher Scientific, Waltham, MA, USA) until total RNA extraction was performed using the High Pure RNA Tissue Kit from Roche (Hoffmann-La Roche, Basel, Switzerland) according to the manufacturer’s guidelines. Reverse transcription was used to synthesize complementary DNA from total RNA.

Relative expression levels of target genes alongside GAPDH (as a reference gene) were quantified using the Roche^®^ LightCycler 480 Thermal Cycler (Roche, Hoffmann-La Roche, Basel, Switzerland) equipped with validated assays ensuring reproducibility and linearity, while employing Invitrogen^®^ (Thermo Fisher Scientific, Waltham, MA, USA) sense and antisense primers along with TaqMan probes from the Universal Probe Library Set (Human from Roche^®^), as detailed in Table 2. All gene expression analyses were conducted in duplicate.

### 4.3. Detecting NDRGs (NDRG1, NDRG2, and NDRG4) in Intestinal Tissue by Immunohistochemistry

Immunohistochemical detection of NDRG1, NDRG2, and NDRG4 was performed on paraffin-embedded intestinal tissues from 10 colectomized patients with severe, treatment-refractory IBD and 10 non-inflamed controls. An expert pathologist conducted all evaluations using predefined criteria. Four μm sections were mounted on electroplated slides, deparaffinized, rehydrated, and subjected to heat-mediated epitope retrieval with DIVA Decloacker (Biocare Medical, Pacheco, CA, USA) for 20 min. Endogenous enzymatic activity was blocked with 3% H_2_O_2_, and nonspecific staining was minimized using a universal blocking solution (Biocare Medical). Tissues were incubated overnight at 4 °C with anti-CD123 (1:50; Santa Cruz Biotechnology, Santa Cruz, CA, USA), followed by MACH4 HRP-polymer and DAB (Biocare Medical) visualization. Additional overnight incubations with anti-NDRG1, anti-NDRG2, or anti-NDRG4 (1:50; Santa Cruz Biotechnology, CA, USA) were developed using the MACH4 AP polymer and Warp Red chromogen (Biocare Medical). Mayer’s hematoxylin was used for counterstaining, and slides were mounted in an aqueous medium. Negative controls employed a universal reagent and a buffer–albumin blank. Because NDGRs are ubiquitously expressed (in most tissues and cells), it is difficult to find tissues that do not express them; however, the antibodies’ specificity was evaluated in adenocarcinoma tissue and in leukocytes, which showed scarce or absent expression. Images were analyzed at 600× magnification.

### 4.4. Statistical Analysis

Statistical analysis was conducted using SPSS software version 22.0 and PRISMA GraphPad package version 6. The data are presented as means ± standard errors of the mean (SEM). For comparisons between independent groups, analysis-of-variance methods were employed alongside Dunn’s tests, and the distribution of variables was evaluated using Shapiro–Wilk tests. The statistical significance threshold was set at *p* < 0.05.

## Figures and Tables

**Table 1:** Clinical and demographic characteristics of IBD patients and controls.

	Active UC (*n* = 20)	Remission UC (*n* = 18)	Active CD (*n* = 10)	Control (*n* = 18)
Median Age (years)	40.3	40.2	41.8	49.4
Sex (Female/male)	8/12	8/10	2/8	13/5
Clinical Course of Disease				
Initially active	6/20	15/18	1/10	
Intermittent	13/20	3/18	5/10	
Continuous	1/20	0/18	4/10	
Extent of Disease				
E1	14/20	9/18		
E2	4/20	5/18		
E3	2/20	4/18		
Extraintestinal Manifestations				
Present	17/20	4/18	8/10	
Absent	3/20	14/18	2/10	
Medical Response to Conventional Treatment				
Favorable response	12/20	17/18	0/10	
No response	8/20	1/18	10/10	

**Table 2:** Characteristics of the primers used in real-time PCR.

Gene	NM Gene Bank	Oligonucleotide Left	Oligonucleotide Right	Universal Probe Library Set
*NR4A1*	NM_001202234.1 NM_001202233.1 NM_173157.2 NM_002135.4	acagcttgcttgtcgatgtc	ggttctgcagctcctccac	UPL#34
*NR4A2*	NM_006186.3	atgaagagagacgcggagaa	aaaagcaatggggagtcca	UPL#63
*NR4A3*	NM_006981.3 NM_173200.2 NM_173199.2	acacccagagatcttgattattcc	gtagaattgttgcacatgctcag	UPL#63
*NDRG1*	NM_001258433.1 NM_001258432.1 NM_006096.3 NM_001135242.1	tcaacgtgaacccttgtgc	gggtccatcctgagatcttg	UPL#42
*NDRG2*	NM_201540.1 NM_201535.1 NM_016250.2 NM_201537.1 NM_201538.1 NM_201539.1	aaaggcaagtgaaggtggaa	ttaggggtcagggttctcact	UPL#74
*NDRG3*	NM_022477.3 NM_032013.3	cctatgtgctggccaagttt	ttggggtcgatgttcacc	UPL#43
*NDRG4*	NM_001242833.1 NM_022910.3 NM_001242834.1 NM_001242835.1 NM_001242836.1 NM_020465.3 NM_001130487.1	cctatgtgctggccaagttt	ttggggtcgatgttcacc	UPL#22
*GAPDH*	NM_002046.3	agccacatcgctcagacac	gcccaatacgaccaaatcc	UPL#60

## Data Availability

The original contributions presented in this study are included in the article. Further inquiries can be directed towards the corresponding author.

## Abbreviations

The following abbreviations are used in this manuscript： Catenin beta 1 gene, CTNNB1; Cluster of Differentiation 123, CD123; Cluster of Differentiation 28, CD28; Crohn’s Disease, CD; C-reactive protein, CRP; Cyclin D1, CCND1; Diaminobenzidine, DAB; Early Growth Response 2, EGR2; Erythrocyte Sedimentation Rate, ESR; F-box protein 11, FBXO11; Glyceraldehyde-3-Phosphate Dehydrogenase, GAPDH; Inflammatory Bowel Disease, IBD; Interleukin-1 BETA, IL-1β; Interleukin-6, IL-6; Interleukin-8, IL-8; N-myc downstream-regulated gene-1, NDRG1; N-myc downstream-regulated gene-2, NDRG2; N-myc downstream-regulated gene-3, NDRG3; N-myc downstream-regulated gene-4, NDRG4; Nuclear Factor kappa B, NF-κB; Nuclear Receptor subfamily 4 group A, NR4A; phosphatidylinositol 3-kinase, PI3K; T cell receptor, TCR; Tumor Necrosis Factor, TNF; Ulcerative Colitis, UC.
